# A Distributed Clustering Algorithm Guided by the Base Station to Extend the Lifetime of Wireless Sensor Networks

**DOI:** 10.3390/s20082312

**Published:** 2020-04-18

**Authors:** Antonio-Jesus Yuste-Delgado, Juan-Carlos Cuevas-Martinez, Alicia Triviño-Cabrera

**Affiliations:** 1 Department of Telecommunication Engineering, Universidad de Jaén, 23700 Linares, Spain; ajyuste@ujaen.es; 2 Department of Electrical Engineering, Universidad de Málaga, 29071 Málaga, Spain; atc@uma.es

**Keywords:** wireless sensor networks, clustering, interval Type-2 fuzzy system

## Abstract

Clustering algorithms are necessary in Wireless Sensor Networks to reduce the energy consumption of the overall nodes. The decision of which nodes are the cluster heads (CHs) greatly affects the network performance. The centralized clustering algorithms rely on a sink or Base Station (BS) to select the CHs. To do so, the BS requires extensive data from the nodes, which sometimes need complex hardware inside each node or a significant number of control messages. Alternatively, the nodes in distributed clustering algorithms decide about which the CHs are by exchanging information among themselves. Both centralized and distributed clustering algorithms usually alternate the nodes playing the role of the CHs to dynamically balance the energy consumption among all the nodes in the network. This paper presents a distributed approach to form the clusters dynamically, but it is occasionally supported by the Base Station. In particular, the Base Station sends three messages during the network lifetime to reconfigure the skip value of the network. The skip, which stands out as the number of rounds in which the same CHs are kept, is adapted to the network status in this way. At the beginning of each group of rounds, the nodes decide about their convenience to become a CH according to a fuzzy-logic system. As a novelty, the fuzzy controller is as a Tagaki–Sugeno–Kang model and not a Mandami-one as other previous proposals. The clustering algorithm has been tested in a wide set of scenarios, and it has been compared with other representative centralized and distributed fuzzy-logic based algorithms. The simulation results demonstrate that the proposed clustering method is able to extend the network operability.

## 1. Introduction

Wireless Sensor Networks (WSN) are a type of network composed of many small, isolated sensors that are distributed in a predetermined area and they communicate with each other via wireless links. A sensor, also called node or mote, is a low-cost, processing device with limited features in terms of computing capacity and energy resources because it is usually powered only with batteries or discontinuous energy sources like solar panels. Advances in the microelectronics and wireless communications make WSN applications very numerous and they are growing continuously [1]. There are WSN applications in many areas such as military, medical, environmental, agricultural, industrial, or smart cities’ environments among others. Some examples of current topics dealing with WSN are in health care [2] or smart grids [3].

**Figure 1:**
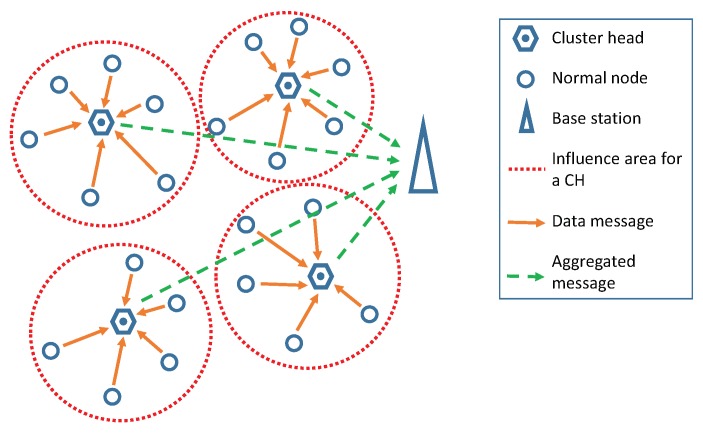
Illustration of a clustering procedure in a wireless sensor network.

The main purpose of those sensors is to monitor some physical variables in their environment and to send their values to a network component that collects all the information to be further processed. This last device is called the sink, gateway, or Base Station (BS). The BS is usually connected to the power grid and it usually has greater computing power, so it does not have the scarce resources of the sensors. Precisely, the power resources in the sensors constitute a limiting characteristic of the WSN as it can prevent the network from operating correctly, that is, gathering all the required information and transmitting it to the BS. Therefore, one of the main challenges of the WSN is to increase the lifetime of the network to avoid nodes depleting their batteries when accomplishing unnecessary tasks. In this sense, one of the most useful techniques is clustering, whereas traditional routing appears better suited for larger networks. A typical example of clustering is found in Figure 1. Among all the nodes, some of them are chosen to become Cluster Heads (CH). The CHs act as gatherers of data of the associated nodes, also referred to as contributing nodes. The nodes transmit their measurements to only one CH, which usually is the one that is closest to them. Then, each CH aggregates the information from its group and relays it to the BS. This technique avoids that all the nodes would have to communicate with the BS directly, which could not be affordable at long distances because they would deplete their battery much faster due to the nonlinear dependence of the power losses with the distance. Additionally, the configuration of the CHs should not be fixed and the nodes should take turns to undertake this function in order to balance the energy consumption of being a CH [4]. Thus, the hierarchy of CHs dynamically changes within the network.

Clustering can be performed in different ways. For those in which CH sends the information directly to the BS (without relaying nodes), the clustering techniques are classified according to the activity performed by the BS. In this set, we distinguish the following clustering strategies:

- Centralized Clustering. The BS has full control about how the clustering is performed. The BS always decides which nodes are converted into CHs. For this operation, the BS needs information about all the nodes in order to choose the most appropriate ones as CHs. The most common properties used for this decision are the location of the node within the sensing area and the residual energy of each node. The first one is not always available in all the applications because nodes usually cannot afford GPS equipment or similar hardware.
- Distributed Clustering. The nodes are completely autonomous and decide by themselves if they become CH or not. This decision is supported by the properties that the node can know/estimate by itself. Then, that information is weighted by some methods that indicate whether to become CH or not. Finally, those nodes that selected themselves as CH send a message to the network so that the other nodes can join them to their clusters.

Centralized and distributed clustering can also be subdivided into different categories depending on the method used to choose the CHs. There are stochastic approaches, geometric algorithms, methods based on fuzzy logic (Type-1 or Type-2), or techniques supported by other artificial intelligence. The most relevant stochastic and distributed algorithms is Low-Energy Adaptive Clustering Hierarchy (LEACH) [5]. LEACH employs a random number that is generated by each node. This number is compared with a parameter representing the probability of becoming CH. The generated value increases with the number of rounds if the sensor has not been chosen as a CH, and it is determined by a system configuration parameter (*p*). If the random number is greater than this value, then the node becomes a CH. An adaptation of this method is detailed in [6], where the nodes add different thresholds depending on the distance or the remaining energy. Other stochastic method is the Hybrid, Energy-Efficient, Distributed clustering approach for ad hoc sensor networks (HEED) [7], a multi-hop clustering algorithm in which the probability of being a CH depends on the residual energy. Normal nodes use the inter-cluster communication cost as a metric to decide which cluster it should join. In an Energy Efficient Clustering Scheme (EECS) [8], the authors propose a clustering method based on a competition among a fixed number of CH candidates. The CH candidates are selected with a probability *T*. This probability is set empirically in a similar way to parameter *p* in LEACH. Other types of approaches are those that combine stochastic and geometrical methods like Voronoi Tessellation. The work in [9] follows this approach for a mobile WSN in which the CH corresponds to the seeds of the cells.

Clustering can also be supported by artificial intelligence. Particularly, Fuzzy Rule-Based Systems (FRBS) outstand as a convenient mechanism to decide which nodes will play the role of the CHs. The applications and research scopes that use these types of expert systems are very numerous in areas such as image classification [10], performance improvement in wind turbines [11], image fusion field [12], software error identification [13], or wireless sensor networks [14]. As for clustering, the work in [15] describes a particle optimization algorithm based on LEACH. The authors of [16] use a fuzzy logic Type-1 distributed algorithm with two outputs. The first output defines the sending radius of the announcement message and the second one determines whether a node will be CH or not.

One of the first relevant centralized methods that employs an expert system is the Cluster-head Election using fuzzy logic (CHEF) [17]. The BS implements a fuzzy logic Type-1 algorithm to decide which node will be cluster head in each round. In [18], the BS selects the best nodes based on a fuzzy Type-2 system. The inputs of this system are the residual energy, the distance to the BS, and the number of neighbors of each node. In the proposal presented in [19], the BS receives information about the energy and the location of the nodes. Then, the BS decides through a simulated annealing algorithm which node configures itself as a CH. Previously, BS has removed as candidates CHs those nodes with an energy below the total average. The work in [20] describes another centralized method that employs a very complex technique. Specifically, it uses a convolutional neural network to determine the best CH. The authors in [21] present a centralized algorithm in which the BS uses artificial-intelligence as fuzzy c-means to determine the best location for the center of each cluster. The authors of [22] show a centralized algorithm that uses a Particle-Swarm based Optimization (PSO) for choosing the best CHs. Additionally, the BS determines the time that a CH remains active based on the residual energy of the system. Although the idea is very promising, the previous implementation is complex in real-time applications because its solution can be only achieved after the convergence of an iterative process.

In this paper, we opt for a hybrid approach which could integrate the benefits of both centralized and distributed clustering algorithms. We propose a mainly distributed algorithm that is guided by the BS at specific instants. In our proposal, the BS sends setup information only three times during the whole lifetime of the network. Thus, those messages change only one characteristic of the distributed algorithm at the following instants: (i) at the beginning of the communication, (ii) when the first node depletes its battery, and (iii) when the network only keeps 50% of alive nodes. This occasional intervention of the BS is easy to implement and results in a better performance. In particular, the messages of the BS are used to dynamically tune the skip value. The operation of a WSN can be divided into rounds. In a traditional approach, at the beginning of each round, the CHs are selected and then the nodes send their data to their corresponding CHs, which will aggregate and forward in a new message to the BS. However, it is possible to maintain the same structure of CHs for several rounds (referred to as the skip value) so that the CH configuration messages are only sent at the first round of this group of skip rounds. This would lead to a reduction of the energy consumption of the CHs. In previous works, the skip value was a constant value [23], but, in this paper, we propose to dynamically adjust it as the inertia of the network varies during its lifetime.

The approach presented in this paper has three main novelties when compared with previous works:

- It is a distributed clustering algorithm guided by the BS in a simple way, with just three transmissions during the network lifetime. The transmissions help the nodes know about the status of the network.
- It uses a dynamic skip value to keep the same CH structure in the network during a skip number of rounds. In this way, the configuration of the skip value is adapted to the dynamic inertia of the network during its operative time. As a consequence, the network can reduce the energy consumption for the CH configuration more efficiently.
- The present paper constitutes a novelty concerning the type of fuzzy system used in the nodes. The nodes implement a new Type-2 Tagaki–Sugeno–Kang (TSK) model for the fuzzy system [24]. Previous fuzzy-logic based clustering algorithms rely on a Mandami model. However, the TSK has revealed itself more appropriate in real-time applications. Moreover, the input variables in the fuzzy system are carefully selected to extend the network lifetime as it is described in Section 2.1.1.

Consequently, this clustering algorithm does not need complex or expensive hardware in the nodes because it is a distributed one. Moreover, it achieves good results without any optimization process in the BS, which could be a demanding process in terms of resources and time. This consumption could not be always negligible, even for the BS. The algorithm has been evaluated in a wide set of scenarios. The results demonstrate that the sporadic intervention of the BS leads to an extension of the network lifetime so that it can operate longer without the battery replacement of the sensing nodes.

The rest of the paper is organized as follows. First, Section 2 presents the main guidelines of the proposed algorithm which encloses the underlying Interval Type-2 Fuzzy System and the Cluster Head election algorithm. Next, Section 3 shows the experiment setup and the results obtained for the comparison with other clustering methods. Finally, the conclusions and future work appear in Section 4

## 2. Proposed Algorithm

In this section, we present the main guidelines of the proposed clustering algorithm. Once the nodes and the BS are deployed, the BS sends the startup message to all the nodes. Nodes use this message to calculate the distance to the BS based on the Received Signal Strength Indicator (RSSI). The network is scheduled to work in a round basis. For each round, nodes run the Interval Type-2 Fuzzy System to evaluate whether they promote themselves as CH or not. Then, those nodes that select themselves as CH send the advice message to the whole network. Non-CH nodes send their data message to the closest CH, which aggregates all the data and relay it to the BS.

In the next sections, first, we explain the underlying Type-2 Fuzzy System that rules the CH selection. Then, we detail the CH selection algorithm that schedules how the network behaves in order to accomplish its application. This description shows the distributed nature of the algorithm, which is occasionally guided by the BS.

### 2.1. Interval Type-2 Fuzzy System

Fuzzy systems are used in numerous proposals about clustering for sensor networks as it behaves properly with the inherent characteristics of these types of networks. This convenience is mainly due to the ability of the fuzzy systems to work on systems with ambiguous, vague, or incomplete inputs [25]. For example, many measurements of a sensor network can be considered incomplete or imprecise. In other situations, the nodes cannot afford to achieve an effective and complete knowledge about their surroundings (other nodes in the network or the environment) because it usually involves the exchange of too many messages. This will saturate the wireless channel and accelerate the depletion of their batteries. In addition, when a node analyzes its own data, non-precise information can be found due to the tolerance of the probes. This happens when evaluating the remaining energy or the distance to the BS that the node computes with the RSSI of different signals.

**Figure 2:**
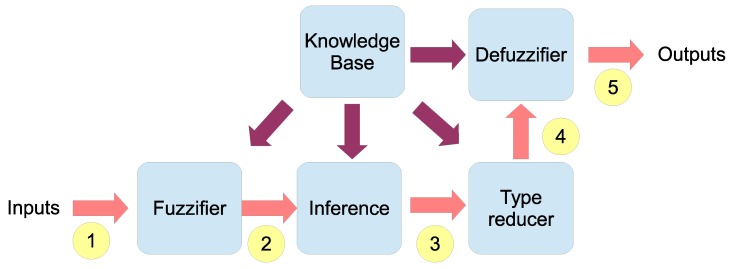
Diagram of a Type-2 Fuzzy System.

In our proposal, we use an Interval Type-2 Fuzzy System (IT2FS) because these types of systems work better than Type-1 ones when the measurements are specially inaccurate or vague [26]. One of the features that makes them suitable to cope with the uncertainty of WSNs is that Type-2 fuzzy systems can incorporate that uncertainty into their fuzzy sets as can be seen in Section 2.1.1. The block diagram of an interval Type-2 fuzzy system, as the one employed in our approach, is presented in Figure 2.

The operation mode of the structure shown in Figure 2 is as follows:

1. The crisp values of the inputs are fuzzified with interval Type-2 fuzzy sets that are stored in the Knowledge Base (KB).
2. Fuzzified inputs are bound together through the rules found in the KB.
3. The inference process binds the inputs, the rules, and the outputs.
4. The interval Type-2 fuzzy is reduced to a Type-1 set by the type-reducer block.
5. The last step is to obtain the output. In our case, the highest and lowest values of the interval are used, each one in a different stage of the process.

Following a distributed clustering approach, each node runs its IT2FS independently and it obtains the output based on its own measurements. To allow an effective deployment and reduce the computational cost of the nodes, the complete solution space of the IT2FS is sampled. Thus, the node only has to quantify the inputs and it gets the right output from a table stored in its memory. This method is much faster than the execution of the inference engine, and we have checked that the error that it introduces is negligible.

Once the main process is described, the system has to be adapted to a specific application (clustering in this case). Therefore, we present the design of the input variables and the layout of the fuzzy sets in the next section.

#### 2.1.1. Variables of the Type-2 Fuzzy System

For this approach, we have employed four input variables and one output variable for the interval fuzzy system. The inputs have been designed taking into account two fundamental aspects related to the lifetime of the network. First, two inputs are related to the energy of the nodes. As a consequence, as much energy a node has, it is more likely to be considered as a cluster head. Second, the other two inputs are based on the performance of a node when it acts as a cluster head. The four inputs used are described as follows:

- $E_{r}$. Remaining energy. This variable measures the percentage of energy that a node has with respect to its initial one. Thus, it ranges from 1 (full battery) to 0 (empty battery). If this value is low, the probability of the node of being chosen as CH should be smaller.
- $N_{CHr}$. This input evaluates the number of times a node has become a CH compared with the total of advice messages of surrounding CHs that it has previously received. $N_{CHr}$ follows Equation (1). If the node has not been selected as a CH for a long time, this value will be high hence. As a consequence of this, the probability of becoming CH will increase:
  (1) $$N_{CHr}=1-\frac{N_{CH}}{N_{total}}.$$
  where $N_{CH}$ is the number of times that the node has been selected as CH and $N_{total}$ is the total number of advice messages received from other CH since the network started to operate.
- $E_{rCH}$. It is the energy that the node has when compared with the average energy of all the nodes that were selected as CHs in the previous round. Consequently, each time a node becomes a CH, it includes its remaining energy in the advice message and then each node computes the average of all the values received from the CHs in the previous iteration to obtain this value. The value is normalized according to Equation (2):
  (2) $$E_{rCH}=\left\{\begin{array}{ccc} 1 & if & E\geq 1.5\;mean(E_{CH})\\ \frac{E}{1.5\;mean(E_{CH})} & if & E<1.5\;mean(E_{CH}) \end{array}\right.$$
  where *E* is the energy of the node and $mean(E_{CH})$ is the average of the energy values received in the advice messages in the preceding iteration. This message is sent to the entire sensing area, so it reaches all nodes. The election of the value 1.5 for the normalization parameter of the threshold in the equation is due to the need for limiting the range of the variability of $E_{CH}$. We have empirically determined that setting $E_{CH}$ to a value greater than $1.5mean(E_{CH})$ does not affect the results significantly. We also observed that the proposed setting also reduces the fluctuation of the system. Alternatively, a node that has been chosen as a CH in a previous round is very likely to have lower energy than those nodes which have only been sensors. In this case, this value will be small, and it would not be chosen again as CH.
- $R_{noCHr}$. It is a function of the number of rounds past since a node became CH. It is calculated as indicated in Equation (3):
  (3) $$R_{noCHr}=\left\{\begin{array}{ccc} 1 & if & R\geq N_{nodes}\\ \frac{R}{N_{nodes}} & if & R<N_{nodes}) \end{array}\right.$$
  where *R* is the number of rounds since a node has not been CH and $N_{nodes}$ is the total number of sensors in the network. If a node has not acted as CH for a long time, this variable will have a high value, which will try to increase the output of the IT2FS. This input prevents a node from remaining as a contributing sensor for a long time.

**Figure 3:**
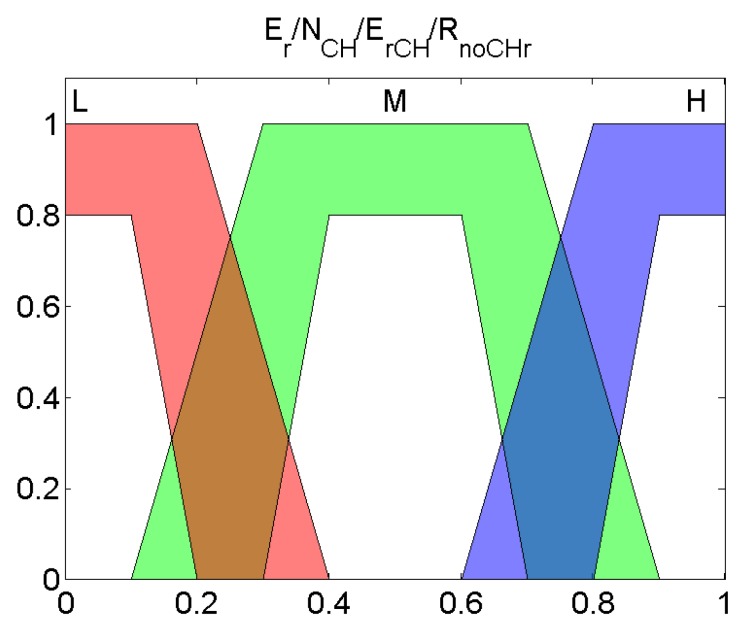
Input Type-2 Fuzzy Set.

Those four inputs are fuzzified into three interval Type-2 sets as shown in Figure 3. The three fuzzy sets are: (i) “low” (L), (ii) “medium” (M), and (iii) “high” (H). Each input value is fuzzified with those sets whose design overlaps between them.

The inference engine uses the rules stored in the knowledge base (see Section 2.1.2) to obtain the output membership function that is based on the fuzzified values of the input variables commented above. In contrast to our previous fuzzy-based clustering algorithms, we propose that the inference process follows a Tagaki–Sugeno–Kang (TSK) model [24]. Consequently, the output variables, as in other TSK systems, are functions whose parameters are the input variables. Those functions are usually linear combinations or constants. The reason for using TSK instead of Mamdani [27] is because, in TSK systems, the consequent of each rule can have as many parameters per rule as input values. This assures more degrees of freedom in the design than a Mamdani one and it provides more flexibility in the design of the system [28]. Another important feature of TSK systems over Mamdani ones is that they can approximate any Mamdani system with an arbitrary level of precision [29]. Moreover, because TSK rule’s consequent is formed by a combination of parameters, TSK systems are more suitable than Mamdani systems in applications that use adaptive or optimization techniques, since it is more efficient [30]. The output intervals in our proposal, noted as ($o_{l}$, $o_{h}$), are formed by five output sets configured as constants as it is detailed in Table 1:

#### 2.1.2. Knowledge Base of the Type-2 Fuzzy System

The knowledge base is composed of a set of IF-THEN clauses that bind all the inputs and the output to encompass the expert knowledge about the application. An example of a rule is as follows:

IF $E_{r}$ is Low AND $N_{CHr}$ is Low AND $E_{rCH}$ is Low AND $R_{noCHr}$ is Low THEN output is Very Low.

This example rule encloses a basic expected behavior: if a node has a low battery level, its relative energy is low, and it has been selected as a CH several times recently, its probability of being chosen as CH should be very low to avoid becoming again a CH and to allow a better energy balance in the network.

Additionally, taking into account that the system is a TSK, we can freely adjust the output variable to suit the requirements of the systems (see Table 1), whereas, with a Mamdani system, the output values are not predictable or adjustable beforehand.

For our implementation, the knowledge base is composed of 81 rules that are displayed in Table 2.

### 2.2. Skip Value Setup

In distributed clustering methods, the node operation that demands more energy is announcing to others that a node has become a CH. This operation should require that the advice message reaches every corner in the deployment field, which usually implies long distances. After each CH sends the advice message, all the contributing nodes choose the closest CH based on the RSSI and they send their data to it. Consequently, as less advice messages are sent, the longer the network lifetime will be. In our approach, to improve the effective lifetime of the network, the nodes that have been promoted themselves as CH will remain in this situation for a certain number of rounds, avoiding sending new advice messages while this configuration is kept. This decision is taken so that the expense due to the advice message is distributed in several rounds. The number of rounds in which the same configuration of CHs is maintained is referred to as skip. The convenient value for the skip should be set according to the observed inertia of this type of network. However, this skip parameter should not be very high because the CHs would deplete their energy quickly due to the amount of data that must be sent to the BS. Nevertheless, the skip parameter should not be too low because it would have no effect to extend the network lifetime [31].

Therefore, in order to tune this parameter according to the evolution of the network, we present a new dynamic skip parameter setting guided by the BS. Thus, the BS is responsible for changing this value three times by sending a setup message at three different instants:

- At network startup. The BS sends a message for the nodes to start working. In that message, it will include the initial value of skip or $skip_{init}$. This message allows each node to estimate the distance to the BS based on the RSSI of the received signal.
- After the first death of a node. To detect that instant, each time a CH sends information to the BS, it includes the number of its contributing nodes. Consequently, the BS, after receiving all the CH data messages, can detect if the total number of nodes that sent information is lower than the initial one. If this happens, the BS will assume that there is one dead node at least. Thus, when it detects the first death of a sensor, it will send a new configuration message with a new value of the skip parameter or $skip_{FND}$.
- After half of the nodes are dead. When the BS detects this event, it configures the nodes with $skip_{HND}$. This value will be kept constant until the network stops operating.

The two messages after the initial setup: (i) after the first dead and (ii) when the number of nodes is halved, have been chosen carefully based on the previous research and empirical analysis. Both are tied to those important moments because they mark the tendency of the network lifetime. First, when the first node dies, some other nodes could have little residual energy too due to their work as CH in a previous round. Hence, the skip value should decrease to allow other nodes to become CH and balance the global residual energy. Thus, if the second setup message is sent too early, the first value of the skip, which is the highest, will lose effectiveness because, when nodes have higher energy, this is when CHs can be kept unchanged longer. The third message is sent when half of the nodes are dead so it is possible that many nodes are close to death. Consequently, the skip value is set to 2 to select each two rounds a new set of CHs, which is proven to be a good solution as we stated in [23]. If the second message is delayed to any other instant after the 50% of the deaths, it could achieve minor benefits because the skip value is greater than 2. In some scenarios, where nodes are grouped in some small areas, delaying the second message would also deteriorate the energy balance of nodes in those areas.

The skip values used in this initial proposal are included in Table 3. These values have been chosen based on the *p* parameter of the well-known algorithm LEACH. This parameter is considered as the optimal percentage of CHs in a WSN and its value is usually set to 0.05. Thus, to obtain the optimal number of CHs in a network, the parameter *p* is multiplied by the total number of nodes. The number of skip parameters is limited to three because it represents the three main networks stages with an important variation of the number of available nodes.

In summary, at the beginning, when all the nodes are available, the skip value is higher due to the redundancy of nodes and the amount of battery they have. The second stage begins when a node dies, which usually implies that an important number of nodes are close to death. As a consequence, the skip value is reduced to change the selected CHs more often and redistribute the energy consumption caused by being a CH among the remaining nodes. Finally, when only 10% of the nodes are available, this skip value s kept at a minimum value to redistribute the CHs between all the alive nodes frequently, but not at every round.

The next section details the cluster head selection algorithm that is run every skip round to rearrange the pool of active CHs.

### 2.3. Algorithm for the Cluster Head Election

The election algorithm presented in Algorithm 1 rules the process of every node to select itself as a CH. After receiving any message from the BS, all the nodes update their skip value and it obtains the output of the Type-2 Fuzzy System ($o_{l}$, $o_{h}$). This output is mapped in persistent memory, and it is compared with a random number newly generated depending on the node history as can be seen in Algorithm 1. Then, the node that was operating as a CH continues with this role when the random number is lower than or equal to $o_{l}$. If this condition does not hold, the node stops operating as CH. Alternatively, a node that was not working as a CH in the previous selection will become a new CH only when the random number is lower than or equal to $o_{h}$. This process is repeated every skip rounds until a new message from the BS is received or the node depletes its battery.

Once a node selects itself as a CH, it sends a message to the entire network. In each round, it receives data from the nodes that are attached to it. Then, it sends the aggregated data to the BS. The CH adds the number of nodes that have been connected to it in that message. It is important to note that the CH structure is maintained for skip rounds and the CH configuration is only sent at the beginning of this set of rounds. In that way, as was stated before, the expensive CH election process is not carried out in each round and we get a trade-off between energy waste, the optimal selection of CHs, and the messages sent. Overall, with this technique, the proposed algorithm achieves a better performance than other representative examples of the bibliography as it is demonstrated in the next section.
**Algorithm 1:** Election of Cluster Head
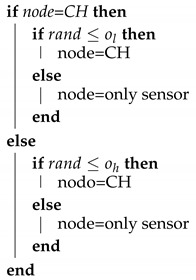


## 3. Evaluation

The proposed clustering algorithm is evaluated by a wide set of simulations, which include several WSN layouts. Those simulations have been carried out over Matlab, which provides a complete suite of mathematical tools. In addition, to acquire valid results, a realistic energy model is included to estimate the energy consumption of the nodes. The details and results of the simulation are presented next.

### 3.1. Energy Model

**Figure 4:**
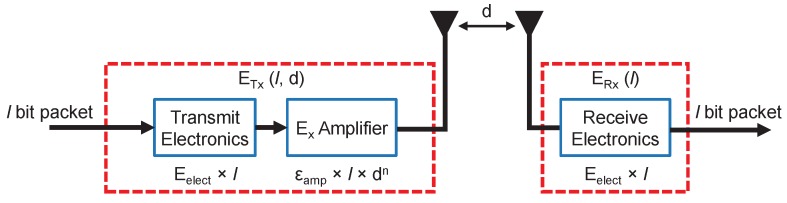
Illustration of the first order radio model.

To carry out the experiments to test the proposed method, an energy model is needed to simulate the data communications by the radio devices. Thus, we use the first order radio model, which is widely used in the related literature [5]. The model, depicted in Figure 4, is described in Equations (4)–(6). To check the results of the energy model, we have compared it with the results presented in [32]. The comparison shows that the results are comparable with the measures shown for a IEEE 802.15.4 compliant radio transceiver CC2420 chip at 2.4 GHz for the average distances employed in our experiments.

(4) $$E_{Tx}\left(l,d\right)=f\left(x\right)=\left\{\begin{array}{cc} l\cdot (E_{elec}+E_{fs}\cdot d^{2}), & d\leq d_{0}\\ l\cdot (E_{elec}+E_{mp}\cdot d^{4}), & d>d_{0} \end{array}\right.$$

(5) $$d_{0}=\sqrt{\frac{E_{fs}}{E_{mp}}}$$

(6) $$E_{Rx}\left(l\right)=E_{elec}\cdot l$$

where:

- *l* is the number of bits of the message.
- $E_{elec}$ is the energy that consumes the transmitter or the receiver circuitry for each bit.
- *d* stands for the distance between the sender and the receiver of the message.
- $E_{fs}$ is the energy consumed by the amplifier according to the free space model $\left(d\leq d_{0}\right)$ to get an acceptable bit error rate (in Figure 4, it appears as $\varepsilon_{amp}$ for both cases).
- $E_{mp}$ is the energy consumed by the amplifier in the multi-path (mp) model $\left(d\leq d_{0}\right)$ to obtain an acceptable bit error rate (in Figure 4 appears as $\varepsilon_{amp}$ for both cases).

When a CH receives data from a contributing node, in addition to the energy spent in receiving the data, the CHs spend energy in the data aggregation process [33]. In the aggregation process, all the CHs send only a summary of those values, which is usually the result of a statistical operation. The aggregation process reduces the number of bits sent when compared with the transmission of each value independently. Therefore, the amount of energy spent by a CH in the reception and aggregation process of a *l*-bit message is defined in Equation (7):

(7) $$E_{Rx-DA}=\left(E_{elec}+E_{DA}\right)\cdot l$$

where $E_{DA}$ is the energy spent by the processing unit of a CH when it aggregates the data received from a contributing node.

### 3.2. Experiment Design

The proposed algorithm is analyzed on a wireless sensor network deployed within a square field of 100 × 100 m${}^{2}$. The number of nodes for all the experiments is 250, which are randomly distributed within the area following a uniform distribution. All the nodes and the BS remain static for the whole network lifetime, and the batteries of the nodes are never charged or replaced. The remaining setup parameters are as follows: the initial energy of all the nodes is 0.5 J, the length of the control messages is 200 bits, and the length of data messages is 2000 bits. All those setup parameters are summarized in Table 4.

**Figure 5:**
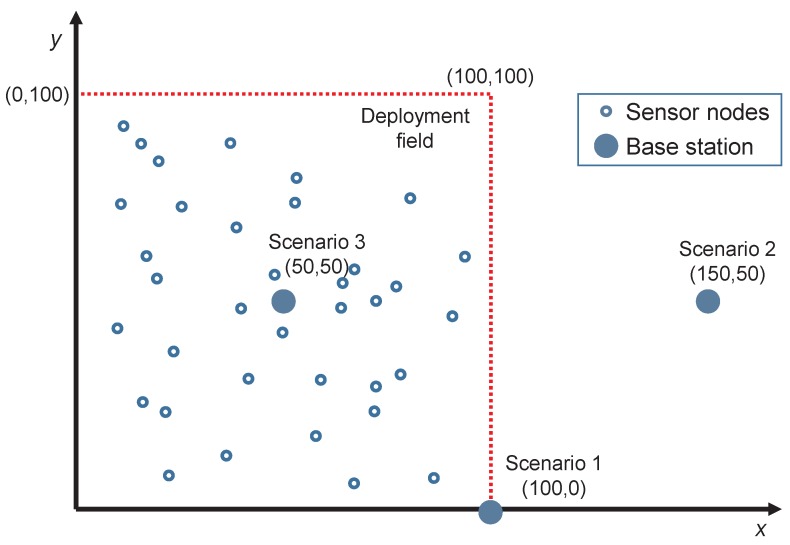
Deployment field for the scenarios used in the experiments.

To evaluate the proposal with different and realistic configurations of WSNs, the BS is placed in three different locations that constitute the three different scenarios to test (see Figure 5):

- Scenario 1. The BS is at one corner of the square field at coordinates (100,0) meters.
- Scenario 2. The BS is far and outside the sensing area at coordinates (150,50) meters.
- Scenario 3. The BS is at the center of the deployment field at coordinates (50,50) meters.

The values for the variables of the energy model, which are the same for all scenarios, are found in Table 5:

Our proposal, named Distributed Clustering Algorithm Guided by Base Station (DCAGBS), is compared with four other proposals widely used in the research literature:

- Low-Energy Adaptive Clustering Hierarchy (LEACH) [5]. It is a distributed algorithm based on a pure stochastic CH selection method which is used like a reference in most clustering proposals. The parameter *p* of this algorithm is set to 0.05 in our simulations.
- Cluster-Head Election using Fuzzy logic for wireless sensor networks (CHEF) [17]. CHEF is a centralized method that decides the best CH based on an expert system. The fuzzy system has three input variables: energy, concentration, and centrality of nodes.
- Energy-Efficient Distributed Clustering algorithm based on a Fuzzy approach with non-uniform distribution (EEDCF) [34]. EEDCF is a distributed algorithm based on fuzzy logic. The inputs are energy, the number of neighbor nodes, and the energy of those nodes. This method has a phase in which different nodes compete to be CH. The node with the best fuzzy output becomes the CH eventually.
- Enhanced Unequal Distributed Type-2 Fuzzy Clustering algorithm (EUDFC) [23]. EUDFC is an interval Type-2 fuzzy distributed system. The system variables are energy, distance to the BS, the average of the distances of the nodes that join a CH, and the number of rounds that a node is only a sensor. EUDFC has a competition phase in which only $n\dot{p}$ CHs is chosen, where *n* is the number of nodes in the system.

In addition to the reference clustering algorithm LEACH, the other algorithms represent illustrative examples of fuzzy-logic based clustering algorithms with a centralized approach (CHEF) and two distributed implementations based on Type-1 and Type-2 Interval Fuzzy systems (EEDCF and EUDFC, respectively).

To test the performance of DCAGBS and the other four methods, they have been evaluated with simulations for the three scenarios. Hence, each scenario has been simulated 30 times with different random node locations to make the results statistically significant. The results obtained from the average of those simulations for each scenario are three metrics widely used in wireless sensor networks: (i) round at which the First, Node Dies (FND), (ii) round at which Half of the Nodes have Died (HND), and (iii) round at which the Last Node Dies (LND). It will be considered that the LND has been reached when only 10% of the nodes (and not for 0%) remain alive because in this situation the network cannot usually operate properly (e.g., acquire the required measurements, transmit the values, etc.) and the exchange of information cannot be considered complete.

### 3.3. Scenario 1

**Figure 6:**
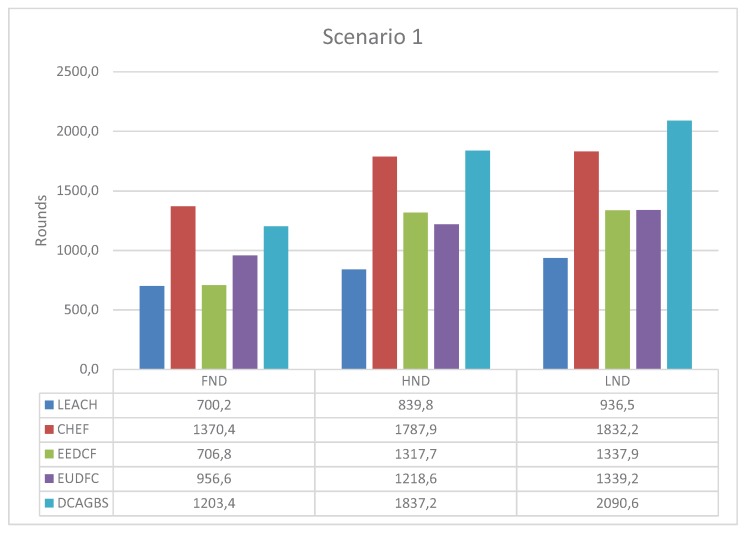
First Node Dead (FND), Half Node Dead (HND), and Last Node Dead (LND) for scenario 1.

In this scenario, the BS is in a corner, which implies that some CHs could be far away from the BS, at the other end of the diagonal of the deployment field. Those further nodes will spend more energy when sending aggregated data to BS. In Figure 6, the plotted data show that CHEF has better values in terms of FND and HND but not in terms of LND. DCAGBS is the best distributed method, and its results are similar to CHEF. In short, DCAGBS adapts very well to this location of the BS, especially for those applications that require a very long lifetime because DCAGBS has the largest LND.

### 3.4. Scenario 2

**Figure 7:**
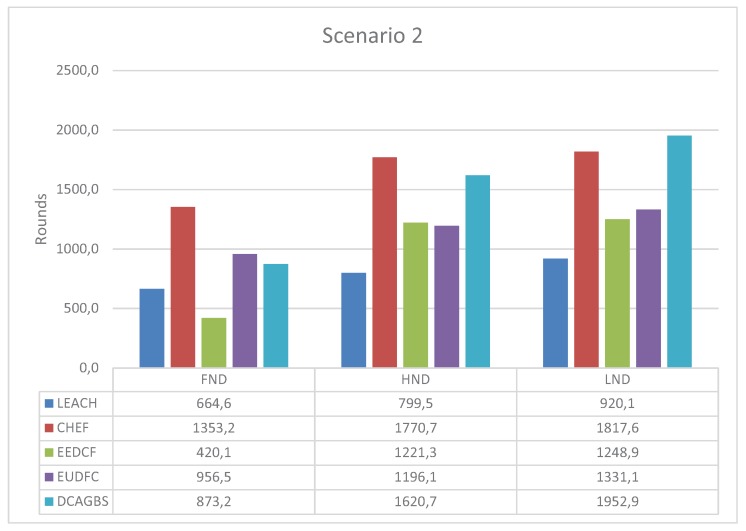
First Node Dead (FND), Half Node Dead (HND), and Last Node Dead (LND) for scenario 2.

The second scenario is the most restrictive one because the BS is outside the sensing area, far from the edge of the network and, consequently, far from all the nodes. Figure 7 shows that the performance metrics are lower than in the previous case for all the tested methods. Again, DCAGBS gets very good results, only matched by CHEF in FND and LND. DCAGBS is the best proposal for LND, which implies that the applications have a longer lifetime. Additionally, the value for HND obtained by DCAGBS clearly outperforms other distributed methods. This implies that, when using DCAGBS, we can get that at least half of the nodes are operative much longer.

### 3.5. Scenario 3

**Figure 8:**
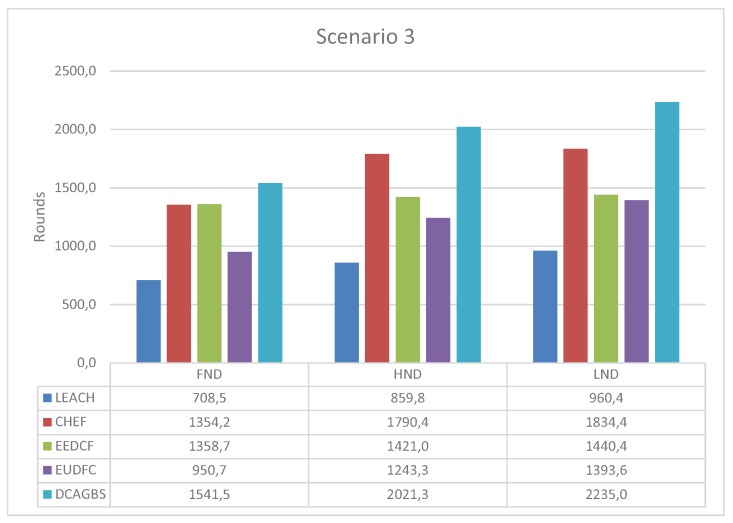
First Node Dead (FND), Half Node Dead (HND), and Last Node Dead (LND) for scenario 3.

The last evaluated scenario is the ideal configuration for a WSN as the BS is placed in the center of the area and the average distance to all nodes is the smallest one Figure 8. Thus, the energy consumption of nodes in each round is lower than in previous scenarios. Consequently, the performance metrics are the highest ones compared with the ones obtained in the other two scenarios. For this configuration, DCAGBS obtains the best results for all metrics compared with all the analyzed algorithms.

### 3.6. Analysis of the Results

We can observe that the hybrid implementation of the clustering algorithm is able to lead to better results for the three types of scenarios because of the dynamic skip value (see Table 3). Thus, in comparison with EUDFC, which employs a constant keep value of 2, DCAGBS with a skip value of 13 (5% of 250 nodes), considerably increases the value of rounds for FND in Scenario 1 and 3. For Scenario 2, due to the long distance to the BS, the value of rounds to the FND are quite similar because the set of CH in DCAGBS are kept constant for a longer time. Then, the number of nodes alive at HND for DCAGBS clearly outperforms EUDFC and EEDCF due to the saved energy in the first period. In the phase from the FND to the HND, which uses the second skip value of 7 (2.5% of 250 nodes), DCAGBS keeps saving energy that will eventually allow it to obtain the best LND for the three scenarios. From the third message, when half of the nodes are dead, all nodes set the skip value to 2. This allows DCAGBS a better balance of the remaining energy in the network while it keeps saving energy. In addition, the input variables in the fuzzy logic system configured for DCAGBS seem to be more appropriate to model the evolution of the network when we compare the results with CHEF.

## 4. Conclusions and Future Work

The distributed clustering algorithms present a series of advantages over the centralized ones in terms of lower cost of the sensors and better adaptation to small scenarios. Centralized algorithms usually get good metrics, but they rely on a vast knowledge of the network, which is not feasible to get in many cases due to the excessive number of messages that should be exchanged or the size, or cost of the additional hardware required in the nodes (e.g., GPS). Our proposal (DCAGBS) presents a BS-guided distributed algorithm that uses an interval Type-2 fuzzy system to adapt the selection of CHs to the changing characteristics of the network like, for example, the lack of accuracy in many control variables. In our method, the BS is able to change the algorithm configuration dynamically. Thus, with the occasional intervention of the BS, the network adapts its configuration according to the death of sensors, changing their behavior after the first and half of the deaths. The algorithm has been compared with other proposals and the goodness of it is demonstrated by simulation in several scenarios. This improvement is especially important in terms of the lifetime of the network, since DCAGBS significantly outperforms all the other proposals.

As future work, we will investigate the proposed approach in larger networks and with a different number of nodes to validate it. Additionally, an optimization of the skip values will be accomplished to see how it is dependent on the new number of nodes and the size of the deployment field. 

## Figures and Tables

**Table 1:** Values for the output interval fuzzy set.

Name	Lower Interval Limit $o_{l}$	Upper Interval Limit $o_{h}$
Very Low	0	0.3
Low	0.2	0.4
Medium	0.3	0.6
High	0.6	0.8
Very High	0.75	1

**Table 2:** Rule base for the Type-2 fuzzy controller.

Rule	Ri	${\mathrm{NCHr}}_{i}$	${\mathrm{ErCH}}_{i}$	RnoCHi	Score		Rule	Ri	${\mathrm{NCHr}}_{i}$	${\mathrm{ErCH}}_{i}$	RnoCHi	Score
1	L	L	L	L	VL		42	M	M	M	H	H
2	L	L	L	M	VL		43	M	M	H	L	M
3	L	L	L	H	L		44	M	M	H	M	H
4	L	L	M	L	L		45	M	M	H	H	H
5	L	L	M	M	L		46	M	H	L	L	L
6	L	L	M	H	VL		47	M	H	L	M	L
7	L	L	H	L	VL		48	M	H	L	H	L
8	L	L	H	M	L		49	M	H	M	L	VL
9	L	L	H	H	L		50	M	H	M	M	L
10	L	M	L	L	VL		51	M	H	M	H	M
11	L	M	L	M	VL		52	M	H	H	L	M
12	L	M	L	H	L		53	M	H	H	M	H
13	L	M	M	L	VL		54	M	H	H	H	VH
14	L	M	M	M	L		55	H	L	L	L	VL
15	L	M	M	H	L		56	H	L	L	M	VL
16	L	M	H	L	VL		57	H	L	L	H	L
17	L	M	H	M	L		58	H	L	M	L	VL
18	L	M	H	H	L		59	H	L	M	M	VL
19	L	H	L	L	VL		60	H	L	M	H	L
20	L	H	L	M	VL		61	H	L	H	L	VL
21	L	H	L	H	L		62	H	L	H	M	VL
22	L	H	M	L	L		63	H	L	H	H	L
23	L	H	M	M	L		64	H	M	L	L	L
24	L	H	M	H	VL		65	H	M	L	M	L
25	L	H	H	L	VL		66	H	M	L	H	M
26	L	H	H	M	VL		67	H	M	M	L	L
27	L	H	H	H	L		68	H	M	M	M	L
28	M	L	L	L	L		69	H	M	M	H	M
29	M	L	L	M	M		70	H	M	H	L	L
30	M	L	L	H	H		71	H	M	H	M	M
31	M	L	M	L	M		72	H	M	H	H	H
32	M	L	M	M	M		73	H	H	L	L	L
33	M	L	M	H	H		74	H	H	L	M	L
34	M	L	H	L	M		75	H	H	L	H	L
35	M	L	H	M	H		76	H	H	M	L	M
36	M	L	H	H	VH		77	H	H	M	M	M
37	M	M	L	L	L		78	H	H	M	H	M
38	M	M	L	M	M		79	H	H	H	L	M
39	M	M	L	H	M		80	H	H	H	M	H
40	M	M	M	L	M		81	H	H	H	H	VH
41	M	M	M	M	M							

Table key: VL = very low, L = Low, M = medium, H = high and VH = very high.

**Table 3:** Skips values.

Skip	Value
$skip_{init}$	5% $N_{nodes}$
$skip_{FND}$	2.5% $N_{nodes}$
$skip_{HND}$	2

**Table 4:** Experiment setup parameters.

Parameter	Value
Deployment field	100 × 100 m${}^{2}$
Nodes deployed	250
Initial energy of nodes	0.5 J
Length of control message	200 bits
Length of data message	2000 bits

**Table 5:** Energy consumption coefficients for the first order radio model and for data aggregation.

Parameter	Value
$E_{elec}$	50 nJ/bit
$E_{DA}$	5 nJ/bit
$E_{fs}$	10 pJ/bit/m${}^{2}$
$E_{mp}$	0.0013 pJ/bit/m${}^{4}$

## References

[1] Mohamed R.E., Saleh A.I., Abdelrazzak M., Samra A.S. (2018). Survey on wireless sensor network applications and energy efficient routing protocols. Wirel. Pers. Commun..

[2] Pike M., Mustafa N.M., Towey D., Brusic V. Sensor Networks and Data Management in Healthcare: Emerging Technologies and New Challenges. Proceedings of the 2019 IEEE 43rd Annual Computer Software and Applications Conference (COMPSAC).

[3] Abujubbeh M., Al-Turjman F., Fahrioglu M. (2019). Software-defined wireless sensor networks in smart grids: An overview. Sustain. Cities Soc..

[4] Liu X. (2012). A Survey on Clustering Routing Protocols in Wireless Sensor Networks. Sensors.

[5] Heinzelman W.R., Chandrakasan A., Balakrishnan H. Energy-efficient communication protocol for wireless microsensor networks. Proceedings of the 33rd Annual Hawaii International Conference on System Sciences.

[6] Kia G., Hassanzadeh A. (2019). A multi-threshold long life time protocol with consistent performance for wireless sensor networks. AEU-Int. J. Electron. Commun..

[7] Younis O., Fahmy S. (2004). HEED: A hybrid, energy-efficient, distributed clustering approach for ad hoc sensor networks. IEEE Trans. Mob. Comput..

[8] Ye M., Li C., Chen G., Wu J. EECS: An energy efficient clustering scheme in wireless sensor networks. Proceedings of the 24th IEEE International Performance, Computing, and Communications Conference.

[9] Pietrabissa A., Liberati F. (2019). Dynamic distributed clustering in wireless sensor networks via Voronoi tessellation control. Int. J. Control.

[10] Huo H., Guo J., Li Z.L. (2018). Hyperspectral Image Classification for Land Cover Based on an Improved Interval Type-II Fuzzy C-Means Approach. Sensors.

[11] Gencer A. (2019). Analysis and Control of Fault Ride-Through Capability Improvement for Wind Turbine Based on a Permanent Magnet Synchronous Generator Using an Interval Type-2 Fuzzy Logic System. Energies.

[12] Jiang Q., Jin X., Hou J., Lee S., Yao S. (2018). Multi-Sensor Image Fusion Based on Interval Type-2 Fuzzy Sets and Regional Features in Nonsubsampled Shearlet Transform Domain. IEEE Sens. J..

[13] Pandey M., Litoriya R., Pandey P. (2019). Identifying causal relationships in mobile app issues: An interval type-2 fuzzy DEMATEL approach. Wirel. Pers. Commun..

[14] Cuevas-Martinez J.C., Yuste-Delgado A.J., Triviño-Cabrera A. (2017). Cluster Head Enhanced Election Type-2 Fuzzy Algorithm for Wireless Sensor Networks. IEEE Commun. Lett..

[15] Moorthi, Thiagarajan R. (2020). Energy consumption and network connectivity based on Novel-LEACH-POS protocol networks. Comput. Commun..

[16] Agrawal D., Pandey S. (2018). FUCA: Fuzzy-based unequal clustering algorithm to prolong the lifetime of wireless sensor networks. Int. J. Commun. Syst..

[17] Gupta I., Riordan D., Sampalli S. Cluster-head election using fuzzy logic for wireless sensor networks. Proceedings of the 3rd Annual Communication Networks and Services Research Conference (CNSR’05).

[18] Zhang F., Zhang Q., Sun Z. ICT2TSK: An improved clustering algorithm for WSN using a type-2 Takagi-Sugeno-Kang Fuzzy Logic System. Proceedings of the 2013 IEEE Symposium on Wireless Technology Applications (ISWTA).

[19] Heinzelman W.B., Chandrakasan A.P., Balakrishnan H. (2002). An application-specific protocol architecture for wireless microsensor networks. IEEE Trans. Wirel. Commun..

[20] Thangaramya K., Kulothungan K., Logambigai R., Selvi M., Ganapathy S., Kannan A. (2019). Energy aware cluster and neuro-fuzzy based routing algorithm for wireless sensor networks in IoT. Comput. Netw..

[21] Shivappa N., Manvi S.S. (2019). Fuzzy-based cluster head selection and cluster formation in wireless sensor networks. IET Netw..

[22] Merabtine N., Djenouri D., Zegour D.E., Boumessaidia B., Boutahraoui A. (2019). Balanced clustering approach with energy prediction and round-time adaptation in wireless sensor networks. Int. J. Commun. Netw. Distrib. Syst..

[23] Yuste-Delgado A.J., Cuevas-Martinez J.C., Triviño-Cabrera A. (2019). EUDFC-Enhanced Unequal Distributed Type-2 Fuzzy Clustering Algorithm. IEEE Sens. J..

[24] Sugeno M. (1985). Industrial Applications of Fuzzy Control.

[25] Zadeh L.A., Klir G.J., Yuan B. (1996). Fuzzy Sets, Fuzzy Logic, and Fuzzy Systems: Selected Papers.

[26] Castillo O., Melin P. (2014). A review on interval type-2 fuzzy logic applications in intelligent control. Inf. Sci..

[27] Mamdani E.H. (1974). Application of fuzzy algorithms for control of simple dynamic plant. Proc. Inst. Electr. Eng..

[28] Jassbi J.J., Serra P.J.A., Ribeiro R.A., Donati A. A Comparison of Mandani and Sugeno Inference Systems for a Space Fault Detection Application. Proceedings of the 2006 World Automation Congress.

[29] Tikk D., Kóczy L.T., Gedeon T.D. (2003). A survey on universal approximation and its limits in soft computing techniques. Int. J. Approx. Reason..

[30] Subhedar M., Birajdar G. (2013). Comparison of mamdani and sugeno inference systems for dynamic spectrum allocation in cognitive radio networks. Wirel. Pers. Commun..

[31] Cuevas-Martinez J.C., Yuste-Delgado A.J., Leon-Sanchez A.J., Saez-Castillo A.J., Triviño-Cabrera A. (2019). A New Centralized Clustering Algorithm for Wireless Sensor Networks. Sensors.

[32] Trakadas P., Zahariadis T., Leligou H.C., Voliotis S., Papadopoulos K. Analyzing energy and time overhead of security mechanisms in Wireless Sensor Networks. Proceedings of the 2008 15th International Conference on Systems, Signals and Image Processing.

[33] Dhand G., Tyagi S. (2016). Data aggregation techniques in WSN: Survey. Procedia Comput. Sci..

[34] Zhang Y., Wang J., Han D., Wu H., Zhou R. (2017). Fuzzy-logic based distributed energy-efficient clustering algorithm for wireless sensor networks. Sensors.

